# A Peculiar New Genus of Bibionomorpha (Diptera) with Brachycera-Like Modification of Antennae from Mid-Cretaceous Amber of Myanmar

**DOI:** 10.3390/insects12040364

**Published:** 2021-04-20

**Authors:** Jan Ševčík, John Skartveit, Wiesław Krzemiński, Kornelia Skibińska

**Affiliations:** 1 Department of Biology and Ecology, Faculty of Science, University of Ostrava, Chittussiho 10, 71000 Ostrava, Czech Republic; 2 Department of Teacher Education, NLA University College Bergen, P.O. Box 74 Sandviken, 5812 Bergen, Norway; John.Skartveit@NLA.no; 3 Institute of Systematics and Evolution of Animals, Polish Academy of Sciences, Sławkowska 17, 31-016 Kraków, Poland; wieslawk4@gmail.com (W.K.); yukisiak@gmail.com (K.S.)

**Keywords:** fossil insects, Bibionidae, inclusions, Mesozoic, taxonomy, new genus, modified antenna

## Abstract

**Simple Summary:**

The mid-Cretaceous amber of Myanmar (also known as Burmese amber) is almost 100 MYA old and represents an invaluable source of information about the evolution of life in the late Mesozoic. This is particularly true for the early flies (lower Diptera) which underwent rapid radiation during the Cretaceous period. Here we describe a remarkable nematocerous fossil fly which shows a character typical of the flies of the suborder Brachycera—with strongly modified antenna—representing new evidence that such a Brachycera-like modification of the antennae has evolved several times during the evolutionary history of Diptera.

**Abstract:**

A new fossil genus of Bibionidae (Diptera: Bibionomorpha), *Burmahesperinus* gen. nov., from the mid-Cretaceous Burmese amber, is described and illustrated (type species *Burmahesperinus antennatus* sp. nov., the other two species included are *B. conicus* sp. nov. and *B. pedicellatus* sp. nov.). The new genus is tentatively placed in a new subfamily, Burmahesperininae subfam. nov. of the family Bibionidae. Its possible phylogenetic position is briefly discussed. The new genus, as well as the subfamily, possesses the wing venation similar to the recent genus *Hesperinus* Walker, 1848, in combination with Brachycera-like modification of both the male and female antenna and the overall habitus typical of fungus gnats (Sciaroidea).

## 1. Introduction

Representatives of the dipteran infraorder Bibionomorpha belong to the most common insects in the fossil record, as well as in recent fauna. There are some 16,500 described species of Bibionomorpha in 34 families and almost 1400 genera, out of which approximately 1400 species are fossil [1,2]. The oldest described fossil taxa assigned to Bibionomorpha *sensu lato* come from the Late Triassic (ca. 220 Mya) of Virginia, USA, and from the Madygen Formation of South Fergana, Kyrgyzstan, dated as Middle or Late Triassic [3,4,5].

Although the phylogenetic interrelationships within the infraorder are far from being clarified [6], Bibionomorpha in its strictest sense (Bibionoidea + Sciaroidea) currently comprises 11 extant families plus approximately the same number of exclusively fossil families [1,2,6,7]. These numbers already reflect the inclusion of the former families Lygistorrhinidae and Cascopleciidae into Keroplatidae and Bibionidae, respectively [2,7].

The mid-Cretaceous (Cenomanian) amber of northern Myanmar (Burmese amber) is considered as essential for understanding the origins and diversification of recent families of Sciaroidea [8], and most probably this applies also to the entire infraorder Bibionomorpha. However, apart from the families Mycetophilidae and Keroplatidae, with 16 described species in total [8,9], little attention has been devoted to the study of the other taxa of Bibionomorpha in Burmese amber and in the Cretaceous as a whole. In contrast, there are numerous taxa of Bibionomorpha described from Jurassic compression fossils [10,11]. Most recently, a new lower Cretaceous subfamily, Cretobibioninae Skartveit and Ansorge 2020, was established [12], representing one of the few recently described Cretaceous taxa of Bibionidae. The other example is the Burmese amber genus *Cascoplecia* Poinar, 2010 [13], initially described in a separate family but later transferred by Pape et al. 2011 [2] to Bibionidae.

The concept of the family Bibionidae differs among authors. North American authors [14,15] have usually preferred a traditional broad concept of Bibionidae, i.e., including the subfamily Hesperininae, while European authors mostly prefer narrower concepts of the families [16], recognizing the separate family Hesperinidae and sometimes even Pleciidae [17]. Actually, most subfamilies in Bibionidae *sensu lato* (with the exception of Bibioninae) are monogeneric and their synapomorphies are the same as for the genera included. Diagnoses, descriptions, and morphological synapomorphies of all extant genera of Bibionidae were given by Fitzgerald [18] and Pinto and Amorim [19].

In this paper, we describe a new fossil genus of Bibionomorpha possessing a very unusual feature—modified antennal flagellum in both sexes—a feature typical for the flies of the suborder Brachycera, with the only other examples hitherto known within the lower Diptera (Nematocera) being two genera of the fossil family Archizelmiridae [20]. We tentatively place this new genus in a new subfamily of Bibionidae.

## 2. Materials and Methods

Specimens were examined using a Nikon (Minato, Japan) SMZ25 stereomicroscope equipped with a Nikon DS-Ri2 digital camera. Photomicrographs are focus stacks captured using this system and processed using NIS-Elements Imaging Software. Line drawings were produced by tracing photographs or with a drawing apparatus attached to the microscope. The terminology principally follows that of Fitzgerald [15] and Skartveit [16], while the terms of wing venation are mainly after Krzemińska et al. [21]. For the explanation of the wing vein nomenclature used in this paper, see the last paragraph of the Discussion below. All the holotypes come from the Hukawng Valley in Kachin State [22,23], northern Myanmar, and they are deposited in the Institute of Systematics and Evolution of Animals, Polish Academy of Sciences, Kraków, Poland (ISEA PAS). Burmese amber is dated to the earliest Cenomanian (98.79 ± 0.62 Ma), based on U-Pb dating of zircons from the volcaniclastic matrix of the amber [24].

This published work and the nomenclatural acts it contains have been registered in ZooBank, the online registration system for the ICZN (International Code of Zoological Nomenclature). The LSID (Life Science Identifier) for this publication is: LSIDurn: lsid:zoobank.org:pub: 583EB8BF-DD8A-4878-BFC9-23682403E035.

## 3. Results

### 3.1. Systematic Paleontology

Order Diptera Linnaeus, 1758

Infraorder Bibionomorpha Hennig, 1954

Superfamily Bibionoidea Fleming, 1821

Family Bibionidae Fleming, 1821

### 3.2. Description of a New Fossil Material

Subfamily Burmahesperininae subfam. nov.

LSID urn:lsid:zoobank.org:act:A7F7E953-1275-4875-B7A9-CCA0C2AFDBC9

Type genus: *Burmahesperinus* gen. nov.

Type species: Burmahesperinus antennatus sp. nov.

Genera included: *Burmahesperinus* gen. nov.

**Figure 1:**
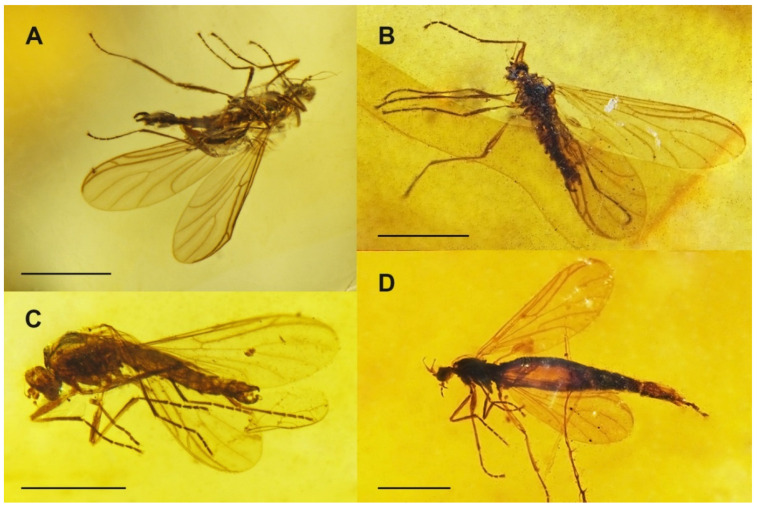
Habitus photographs of *Burmahesperinus antennatus* gen. et sp. nov. ((**A**), holotype), *B. conicus* gen. et sp. nov. ((**B**), holotype), *B. pedicellatus* gen. et sp. nov. ((**C**), holotype), and *Burmahesperinus* sp. ((**D**), female specimen No. MP/4081). Scale bars = 1 mm.

**Figure 2:**
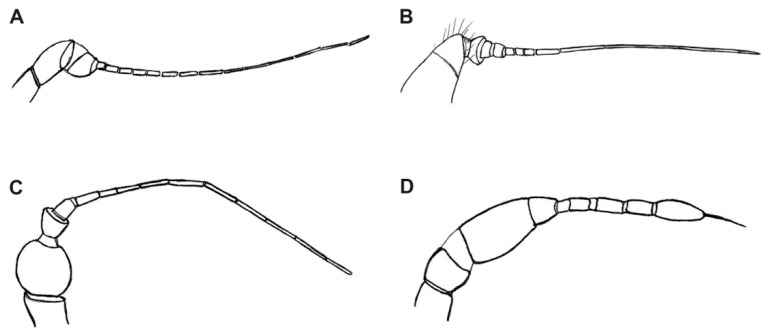
Antenna of *Burmahesperinus antennatus* gen. et sp. nov. ((**A**), holotype), *B. conicus* gen. et sp. nov. ((**B**), holotype), *B. pedicellatus* gen. et sp. nov. ((**C**), holotype), and *Burmahesperinus* sp. ((**D**), female specimen No. MP/4081).

Diagnosis. Relatively small and slender bibionoid flies (Figure 1), resembling both Bibionoidea and Sciaroidea. Body not distinctly hairy. Both male and female antennae about as long as the breadth of the head, flagellum strongly modified, filiform, tapering to the apex. Flagellomeres distinctly differentiated, with the basal segments thicker and the apical segments tapered to a hair-like process (stylus). Wing (Figure 2A) without stigma, with radial veins slightly more chitinized than the other veins. Vein Sc relatively long, ending in C around the middle of the wing and well beyond the level of Rs; R_2+3_ short, not distinctly sinuous; crossvein called bm-m absent (actually basal part of M_3+4_); M_1+2_ long, almost as long as M_2_; A_1_ long but weak, reaching the wing margin. All legs thin and without strong spines, each tibia with a single short tibial spur, not longer than maximum tibial diameter. Coxae short, femora not thickened. Male terminalia with a relatively long gonocoxite, bearing gonostylus about as long as gonocoxite. Gonostylus is simple, apically blunt or with a subapical tooth.

***Burmahesperinus* gen. nov.**

LSID urn:lsid:zoobank.org:act:74A40C8B-E9AE-4DD2-B223-88FDDC6E20F7

Type species: *Burmahesperinus antennatus* sp. nov., by monotypy and present designation.

Diagnosis. The same as for the subfamily.

Etymology. The name of the new genus is composed of Burma (=Myanmar), referring to the origin of the amber, and *Hesperinus*, a similar extant genus.

**Figure 3:**
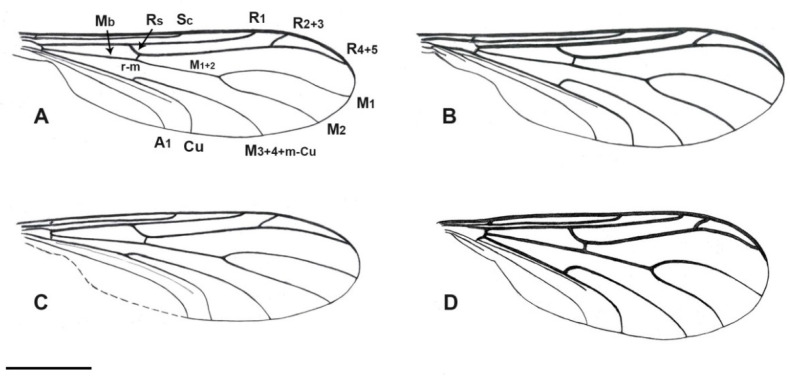
Wing of *Burmahesperinus antennatus* gen. et sp. nov. ((**A**), holotype), *B. conicus* gen. et sp. nov. ((**B**), holotype), *B. pedicellatus* gen. et sp. nov. ((**C**), holotype), and *Burmahesperinus* sp. ((**D**), female specimen No. MP/4081). Abbreviations: Sc, subcostal vein; Rs, radial sector; R_1-5_, branches of radius; r-m, radial-medial crossvein; Mb, medial-basal vein; M_1+2_, stem of media; M_1,2_, branches of media; M_3+4+m-cu_, branches of media fused with crossvein m-cu; Cu, cubital vein; A_1_, first branch of anal vein. Scale bar = 0.5 mm.

**Figure 4:**
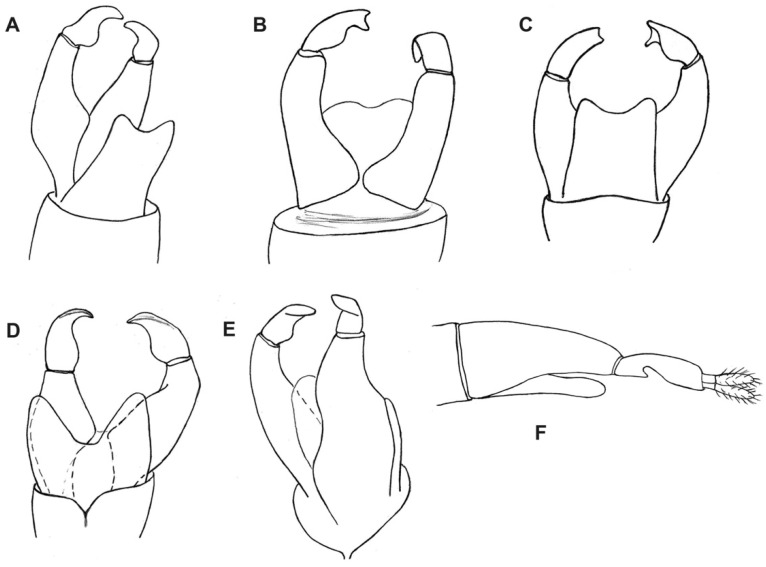
Male and female terminalia of *Burmahesperinus antennatus* gen. et sp. nov. ((**A**), holotype), *B. conicus* gen. et sp. nov. ((**B**,**C**), holotype), *B. pedicellatus* gen. et sp. nov. ((**D**,**E**), holotype), and *Burmahesperinus* sp. (**F**), female specimen No. MP/4081). Dorsal (**A**,**C**,**D**), ventral (**B**) and lateral view (**E**,**F**).

***Burmahesperinus antennatus* sp. nov.** (Figure 1A, Figure 2A, Figure 3A and Figure 4A)

LSID urn:lsid:zoobank.org:act: 4986BA76-962D-41E9-B5E2-81E692CB1BB7

Material. Holotype No. MP/4078, male, in Burmese amber (the earliest Cenomanian, 98.79 ± 0.62 Ma), deposited in the Institute of Systematics and Evolution of Animals, Polish Academy of Sciences, Kraków, Poland (ISEA PAS).

Diagnosis. Scapus and pedicel tubular, almost of the same width; Sc slightly shorter than half of the length of the wing; R_1_ ends in C well before the base of R_2+3_ and is shorter than a half of the wing length; R_2+3_ about 1.5 times as long as Rs.

Etymology. The name *antennatus* refers to the peculiar structure of the male antenna.

Description (male). Body 2.2 mm long; wing length 2.0 mm.

Head relatively small, with eyes widely separated; all ommatidia of the same size. Ocelli present but their details not traceable.

Antenna (Figure 2A) slightly longer than the breadth of the head; scapus long, tubular; pedicel short, tubular, about as broad as scapus; the basal segment of the flagellum wide, short, perhaps formed by the fusion of several segments of the basal flagellomeres, the subsequent segments increasingly thinner and elongated, forming a stylus with only poorly visible boundaries between the segments.

Palpus with four visible palpomeres of similar length; all of them thin and relatively long.

Thorax slightly arched, about 1.5 times as long as high. Scutum and lateral sclerites mostly bare, without distinct hairs or spines.

Wing (Figure 3A): 3.2 times as long as wide. Wing membrane hyaline, without macrotrichia and without stigma or other markings. Radial veins relatively strong and thickened; C ends beyond the apex of R_5_, reaching to about a third of the distance between the apex of R_4+5_ and M_1_; Sc ends in C just before the half of the wing length; R_1_ ends in C well before the base of R_2+3_; R_2+3_ about 1.5 times as long than Rs; R_4+5_ almost four times as long as R_2+3_; r-m at the level of the base of M_3+4+m-cu_; vein M_3+4+m-cu_ by 1/3 longer than the section of Cu located beyond the base of M_3+4+m-cu_; cell M_3+4_ approximately 1.25 as wide as M_2_ cell.

Legs relatively long, thin, with the coxae short and femora at least ten times as long as broad. Legs covered with dense short setae, without any strong spines. Each leg with a single tibial spur, not longer than maximum tibial diameter.

Abdomen sparsely covered with relatively short hairs.

Male terminalia (Figure 4A) as in most Bibionidae, except for the longer and narrower gonocoxite. Tergite 9 relatively short, about twice as broad as long, with a slightly cleft posterior margin (external); cerci about as long as gonocoxites, bearing long thin setae; gonocoxite broad at the base, sickle shaped and tapering towards the apex, with a clear extension in the basal part and additionally extended in 1/3 of its length; gonostylus wide, plane-like, tapering towards the apex. Internal structures of terminalia are not traceable in the holotype.

***Burmahesperinus conicus* sp. nov.** (Figure 1B, Figure 2B, Figure 3B and Figure 4B,C)

LSID urn:lsid:zoobank.org:act: 5526B751-8148-46BA-8513-79BDA6CB5E52

Material. Holotype No. MP/4079, male, in Burmese amber (the earliest Cenomanian, 98.79 ± 0.62 Ma), deposited in the Institute of Systematics and Evolution of Animals, Polish Academy of Sciences, Kraków, Poland (ISEA PAS).

Diagnosis. Scapus and pedicel apparently fused, forming a uniform conical structure narrowing towards the apex; Sc slightly longer than half the length of the wing; R_1_ ends in C almost at the level of the base of R_2+3_; R_2+3_ about as long as Rs.

Etymology. The name *conicus* refers to the conical shape of the fused basal segments of the male antenna.

Description. Body 1.8 mm long; wing length 2.3 mm.

Head relatively small, with eyes widely divided; all ommatidia of the same size. Ocelli present but their details not traceable.

Antenna (Figure 2B) slightly longer than the width of the head; scapus and pedicel joined, forming a uniform structure wide at the base and narrowing towards the apex; the first flagellomere broad, bowl-shaped, short and perhaps formed by the fusion of several segments of the basal flagellomeres, the subsequent flagellomeres are thinner and more elongated, forming a flagellum with poorly visible boundaries between the segments.

Palpus with four visible palpomeres of similar length; all of them thin and relatively long.

Thorax slightly arched, about 1.5 times as long as high. Scutum and lateral sclerites mostly bare, without distinct hairs or spines.

Wing (Figure 3B): 3.1 times as long as wide. Wing membrane hyaline, without macrotrichia and without stigma or other markings. Radial veins relatively strong and thickened; C ends slightly beyond the apex of R_5_, reaching to about 1/4 of the distance between the apex of R_4+5_ and M_1_; Sc longer than half of the wing length; R_1_ ends in C almost at the level of the base of R_2+3_; R_2+3_ about as long as Rs; R_4+5_ almost four times as long as R_2+3_; r-m clearly beyond the base of M_3+4+m-cu_; vein M_3+4+m-cu_ about 1/3 longer than the section of Cu located beyond the base of M_3+4+m-cu_; cell M_3+4_ slightly narrower than cell M_2_.

Legs relatively long, thin, covered with setae, each leg with a single tibial spur, about as long as maximum tibial diameter.

Male terminalia (Figure 4B,C) as in most Bibionidae, except for the longer and narrower gonocoxite. Tergite 9 relatively short, about twice as broad as long, with a slightly cleft posterior margin (external); cerci about as long as gonocoxites, bearing long, thin setae; gonocoxite broad at the base, sickle shaped, basally broad but without any additional extension in 1/3 of its length; gonostylus wide, plane shaped, with a distinct fork apically. The gonostyli appear as asymmetrical in the type specimen, with the left and right gonostylus different apically (Figure 4C). Internal structures of terminalia are not traceable in the holotype.

***Burmahesperinus pedicellatus* sp. nov.** (Figure 1C, Figure 2C, Figure 3C and Figure 4D,E)

LSID urn:lsid:zoobank.org:act: 96E70BF5-DB94-40E4-A631-9689CCF791E9

Material. Holotype No. MP/4080, male, in Burmese amber (the earliest Cenomanian, 98.79 ± 0.62 Ma), deposited in the Institute of Systematics and Evolution of Animals, Polish Academy of Sciences, Kraków, Poland (ISEA PAS).

Diagnosis. Scapus long, tubular; pedicel relatively large, spherical; Sc much shorter than half the wing length; R1 ends in C well before the base of R_2+3_; R_2+3_ only slightly longer than Rs.

Etymology. The name *pedicellatus* refers to the unusual spherical shape of the pedicel.

Description. Body 2.3 mm long; wing length 2.1 mm.

Head relatively small, with eyes widely divided; all ommatidia of the same size. Ocelli present but their details not traceable.

Antenna (Figure 2C) slightly longer than the width of the head; scapus long, tubular; pedicel very large, globular; the first flagellomere broad, bowl shaped, short, the subsequent parts are thinner and more elongated, forming a flagellum with poorly visible boundaries between the segments.

Palpus with four visible palpomeres of similar length; all of them thin and relatively long.

Thorax slightly arched, about 1.5 times as long as high. Scutum and lateral sclerites mostly bare, without distinct hairs or spines.

Wing (Figure 3C): 3.1 times as long as wide. Wing membrane hyaline, without macrotrichia and without stigma or other markings. Radial veins relatively strong and thickened; Sc significantly shorter than half of the wing length; ends in C well before the base of R_2+3_; R_2+3_ is only slightly longer than Rs; R_4+5_ almost four times as long as R_2+3_; r-m is well beyond the base of m-cu+M_3+4_; m-cu+M_3+4_ twice as long as the section of Cu located beyond the base of m-cu+M_3+4_; cell M_3+4_ equals the width of the opening of the cell M_2_.

Legs relatively long, thin, covered with setae, each leg with a single tibial spur, about as long as maximum tibial diameter.

Male terminalia (Figure 4D,E) as in most Bibionidae, except for the longer and narrower gonocoxite. Tergite 9 relatively short, about twice as broad as long, with a deeply cleft posterior margin; cerci about as long as gonocoxites, bearing long thin setae; gonocoxite narrow basally, clearly extended in the middle of its length; gonostylus broad basally, flattened, narrowing towards the end. Internal structures of terminalia are not traceable in the holotype.

***Burmahesperinus* sp. (female)** (Figure 1D, Figure 2D, Figure 3D and Figure 4F)

Material. Specimen No. MP/4081, female, in Burmese amber (the earliest Cenomanian, 98.79 ± 0.62 Ma), deposited in the Institute of Systematics and Evolution of Animals, Polish Academy of Sciences, Kraków, Poland (ISEA PAS).

Description. Body 3.8 mm long; wing length 2.2 mm.

Similar to male in most characters.

Head relatively small, eyes dichoptic, widely separated, all ommatidia are of the same size. Ocelli placed on a prominent medial tubercle. Antenna (Figure 2D) strongly modified, tapering to the apex, shorter than the width of the head; scapus tubular; pedicel short; basal section of the flagellum large, broad, broader than the width of the pedicel, closely connected to the second section of the flagellum in the shape of an inverted bowl, and the next three sections narrow and rather short, while the last (sixth) section of the flagellum clearly extended and longer than the penultimate one with a long bristle at the end; palpi four-segmented, the last one over twice as long as the penultimate one.

Thorax slightly arched, about 1.5 times as long as high. Scutum and lateral sclerites mostly bare, without distinct hairs or spines.

Wing (Figure 3D): 2.7 times as long as wide. Wing membrane hyaline, without macrotrichia and without stigma or other markings. Radial veins relatively strong and thickened; Sc slightly longer than half of the wing length; R_1_ ends in C well before the base of R_2+3_; Rs almost 1.5 times as long as R_2+3_; R_4+5_ almost four times as long as R_2+3_; r-m is well beyond the base of M_3+4+m-cu_; M_3+4+m-cu_ almost twice as long as the section of Cu located beyond the base of M_3+4+m-cu_; cell M_3+4_ distally about 1.3 as wide as the opening of the cell M_2_.

Legs relatively long, thin, covered with setae, each leg with a single tibial spur, about as long as maximum tibial diameter.

Abdomen elongated and swollen in basal half.

Terminalia as in Figure 4F.

Remarks. Currently, it is not possible to assign this female unequivocally to any species described here. This female shows a different way of the reduction and overall structure of the antennae than in the males described above. Perhaps it is another example of sexual dimorphism in Bibionidae, or the described female represents yet another unknown species of *Burmahesperinus* gen. nov. This problem can possibly be solved in the future with more specimens available. The wing venation may suggest this might be the female of *B. conicus* sp. nov. The features present in the latter species but different from those in both *B. antennatus* sp. nov. and *B. pedicellatus* sp. nov. include medial portion of Cu and proximal half of M_3+4+m-cu_ parallel (diverging in the other two species) and the fork of M_1_ and M_2_ basally more rounded (narrow in the other two species). We prefer, however, to leave this female unassigned to a species, until more material is available and possible variation in wing vein characters is better known.

### 3.3. Identification Key to Species of Burmahesperinus gen. nov.

1. Sc longer than half the wing length; R_1_ ends in C almost at the level of the base of R_2+3_. Scapus and pedicel fused, forming a uniform conical structure ……………………………………………………………………………… *B. conicus* spec. nov.

Sc shorter than half of the wing length. R_1_ ends in C before the base of R_2+3_ ……… 2

2. Scapus and pedicel tubular, almost of the same width ........ *B. antennatus* spec. nov.

Scapus long, tubular; pedicel spherical ................................... *B. pedicellatus* spec. nov.

## 4. Discussion

A similar pattern of wing venation like in *Burmahesperinus* gen. nov. can be seen in the genus *Hesperinus* Walker, 1848, a genus based on the extant type species but known also from Baltic amber [25] or in several genera of the fossil family Pleciofungivoridae [10,11], e.g., in *Eohesperinus* Rohdendorf, 1946 or *Pleciofungivora* Rohdendorf, 1938. In all these taxa, however, the vein R_2+3_ is substantially longer and sinuous, and the crossvein bm-m (according to [26] or bm-m sensu [16], actually the basal part of M_3+4_) is well developed, regardless of the typical nematoceran structure of the antennae. In extant species of *Hesperinus*, a distinct pterostigma is also present (although probably absent in some of the Baltic amber representatives [25]) and the stem of M-fork (M_1+2_) is distinctly shorter (while almost as long as M_1_ in *Burmahesperinus*). The extant species of *Hesperinus* are also substantially larger, with wing length being around five times as long as that of *Burmahesperinus*. On the other hand, both *Burmahesperinus* and *Hesperinus* share the narrow wing shape, with little developed anal lobe, dichoptic eyes in male (while the male eyes are holoptic in all the other extant Bibionoidea), and reduced mouthparts.

Additionally, several primitive extant genera of Sciaroidea show some similarities to *Burmahesperinus* in the ground plan of the wing venation, as well as in the relatively simple male terminalia, for example *Arachnocampa* Edwards, 1924; *Bolitophila* Meigen, 1818; and *Catotricha* Edwards, 1938, but proportions of particular veins (including the presence or absence of the crossvein bm-m sensu [26]) are different among these taxa, regardless of some other body characters typical of Sciaroidea. In particular, in most Sciaroidea (except of Cecidomyiidae), both the coxae and tibial spurs are much longer than in *Burmahesperinus*. However, in the broad and heterogeneous groups like Sciaroidea, unique synapomorphies defining the entire group are difficult or impossible to find, so the concept of these suprageneric taxa may differ among particular authors. This may potentially complicate the systematic placement of some peculiar fossil taxa, including *Burmahesperinus*. In any case, we do not prefer to place *Burmahesperinus* in Sciaroidea.

We have decided to place the new genus in the Bibionidae sensu lato, based mostly on the highly plesiomorphic wing venation similar to that in *Hesperinus*. However, its placement in the separate new subfamily Burmahesperininae should be considered as tentative, pending new discoveries in the fossil record, especially in the Mesozoic, which may change the concept and position of the new subfamily. Fitzgerald [18] suggested the complete postgenal bridge as a synapomorphy for Bibionidae but this character state is not traceable in most fossil specimens, including the type material of *Burmahesperinus*.

However, the most striking feature of the new genus is the strongly modified antenna, both in male and female. Within the lower Diptera (Nematocera), a similarly reduced antennal flagellum is present only in two genera of the entirely fossil family Archizelmiridae, viz. *Burmazelmira* [18] from Burmese amber and the Spanish Lower Cretaceous (upper Albian) amber [27] and *Archimelzira* [18] from the upper Cretaceous (Turonian) amber of New Jersey [18]. The antennal flagellum of *Burmazelmira* shows the highest degree of modification (stylus represents more than three distal quarters of the length of the flagellum), hitherto a unique example in the order Diptera outside the suborder Brachycera [18]. However, many other characteristics, including the shape of wing and wing venation (especially the quite different position of veins in the proximal half of a wing), rule out a possible placement of *Burmahesperinus* gen. nov. in Archizelmiridae.

Evidently, such a modification of the antennae has evolved several times during the evolutionary history of lower Diptera, and *Burmahesperinus* gen. nov. is another illustration of this process. Numerous examples in modern fauna, as well as in the fossil record, show that the reduction and transformation of the antennae from the primitive multi-segmented type found in lower Diptera to the structure known in most Brachycera has occurred repeatedly, as Stuckenberg [28] extensively describes in examples of lower Brachycera. On the other hand, the opposite case of the flagellum with 14 fully developed flagellomeres is known in several taxa of lower Brachycera, e.g., in the genus *Rachicerus* Walker, 1854 (family Rachiceridae), where the male flagellum is even pectinate.

Since the number of flagellomeres cannot be exactly documented, we should compare *Burmahesperinus* gen. nov. also with Brachycera to exclude its placement there. Apart from the slender body, long palpi (at least four-segmented) and elongated legs, typical of the lower Diptera (Nematocera), the wing venation is the main argument for the placement of *Burmahesperinus* gen. nov. in Bibionomorpha rather than in Brachycera. Actually, Nematocera is currently considered as a paraphyletic group, hence suggested to be called just lower Diptera, and infraorders of Nematocera are sometimes considered as suborders of Diptera [29]. In any case, both Bibionomorpha and Brachycera represent sister groups, e.g., [6,30], altogether called Neodiptera [31], which means that the closest relative of Bibionomorpha is Brachycera, not the rest of lower Diptera. The new genus definitely belongs to Neodiptera and the modification of the antennae in *Burmahesperinus* may possibly be considered as another indication of the sister relationship of these two major groups of Diptera. Although many currently recognized synapomorphies defining Brachycera [30] are based on the morphology of the thorax, which is rarely preserved enough in fossils to allow seeing its detailed structure, or on larval structures, usually unknown in extinct taxa, we do not see any other characters (except for the modified antennae) in support of a possible inclusion of *Burmahesperinus* in Brachycera.

Another potentially remarkable feature is the asymmetry of the male terminalia, apparent in the holotype of *B. conicus* sp. nov. and also in one more specimen (without antennae) not included in this study. Having just two such specimens available, it is impossible to decide now if this is just an aberration in these specimens, a damage caused by the fossilization process, or a regular feature in this species or genus. We leave this issue as open to future studies because male terminalia of Bibionidae, while symmetrical, can look very different from various angles and in fossil specimens not all views are available. The (real) asymmetry of particular parts of the male terminalia is known to be independently developed in various groups of Diptera, both Nematocera and Brachycera, e.g., in Scatopsidae, Hybotidae, or Dolichopodidae [32,33].

Finally, we present here a short explanation of the wing vein nomenclature used in this paper. We consider Anisopodomorpha as the ancestral form of wing venation for Bibionomorpha. In Anisopodomorpha, there is the medial-basal vein (Mb), which bifurcates into M_1+2_ and M_3+4_. In fact, we do not know if M_3_ disappeared by atrophy or if the veins M_3_ and M_4_ fused along their entire length; therefore, both interpretations may be correct (M_4_ or M_3+4_). In our opinion, it is rather M_3+4_ than M_4_, i.e., the veins fused, based on the observation of wing vein changes in Anisopodomorpha and primitive Bibionomorpha. However, if M_3_ is considered as atrophied along its entire length, then the correct name would be M_4_. In any case, the basal part of either M_3+4_ or M_4_ is the remnant of m-cu, while the vein called bm-m in the Afrotropical Manual [26] is the basal part of M_4_ and in further development of Bibionomorpha, it atrophies. In *Plecia* Wiedemann, 1828, for example, crossvein bm-m equals M_3+4_, and it is actually the posterior wall of the discoid cell, i.e., the basal part of M_3+4_. In some species (as early as in Anispodomorpha), the basal part of M_3+4_ disappears, which can lead to misinterpretation of the veins. In *Burmahesperinus*, Mb forks into M_1+2_ and M_3+4_, and the basal part of the latter disappears (i.e., the fragment called as bm-m [26] disappears), while the end joining Cu is m-cu. Therefore, we think that the entire vein should be called M_3+4_+m-cu (or M_4_+m-cu). This subject will be thoroughly treated in a separate paper under preparation (Krzeminski et al., in prep.).

## 5. Conclusions

The new fossil genus, described in this paper, represents an interesting example of Brachycera-like modification of antennal flagellum within lower Diptera. We place it tentatively in the new subfamily (Burmahesperininae subfam. nov.) of the family Bibionidae, based mainly on the wing venation, pending further studies into the evolution of both recent and fossil Bibionomorpha.
